# Association between maternal alcohol consumption during pregnancy and risk of preterm delivery: the Japan Environment and Children's Study

**DOI:** 10.1111/1471-0528.15899

**Published:** 2019-08-25

**Authors:** S Ikehara, T Kimura, A Kakigano, T Sato, H Iso, the Japan Environment Children's Study Group, Hirohisa Saito, Reiko Kishi, Nobuo Yaegashi, Koichi Hashimoto, Chisato Mori, Shuichi Ito, Zentaro Yamagata, Hidekuni Inadera, Michihiro Kamijima, Takeo Nakayama, Masayuki Shima, Yasuaki Hirooka, Narufumi Suganuma, Koichi Kusuhara, Takahiko Katoh

**Affiliations:** ^1^ Public Health, Department of Social Medicine Osaka University Graduate School of Medicine Suita‐shi Japan; ^2^ Department of Public Health Hokkaido University Graduate School of Medicine Sapporo Japan; ^3^ Department of Obstetrics and Gynaecology Osaka University Graduate School of Medicine Suita‐shi Japan; ^4^ Osaka Maternal and Child Health Information Centre Osaka Women's and Children's Hospital Izumi Japan

**Keywords:** Alcohol consumption, pregnant women, preterm delivery, prospective study, the Japan Environment and Children's Study (JECS)

## Abstract

**Objective:**

To examine the association between maternal alcohol consumption during pregnancy and the risk of preterm delivery.

**Design:**

Prospective cohort study.

**Setting:**

The Japan Environment and Children's Study (JECS).

**Population:**

A total of 94 349 singleton pregnancies.

**Methods:**

Participants completed questionnaires detailing alcohol consumption during the first trimester and during the second and third trimesters. Participants were divided into four categories according to alcohol consumption (non‐drinkers, consumers of 1–149 g, 150–299 g and ≥300 g ethanol/week). We examined the effect of alcohol consumption during different stages of pregnancy on the risk of preterm delivery. Odds ratios (OR) and 95% CI were calculated relative to non‐drinkers using logistic regression.

**Main outcome measures:**

Medical record‐based preterm delivery.

**Results:**

Alcohol consumption during the second and third trimesters, but not during the first trimester, was associated with increased risk of preterm delivery. Heavy alcohol consumption (≥300 g ethanol/week) during the second and third trimesters was associated with a four‐fold higher risk compared with non‐drinkers (multivariable OR 4.52; 95% CI 1.68–12.2). Light alcohol consumption (1–149 g ethanol/week) tended to be associated with lower risk of preterm delivery (multivariable OR 0.78; 95% CI 0.60–1.00).

**Conclusions:**

Heavy alcohol consumption during the second and third trimesters was associated with increased risk of preterm delivery among pregnant women.

**Tweetable abstract:**

Heavy drinking during pregnancy may increase the risk of preterm delivery.

## Abstract 2

Tweetable abstract

Heavy drinking during pregnancy may increase the risk of preterm delivery.

## Introduction

Some cohort studies suggest a J‐shaped association between alcohol consumption and the risk of preterm delivery.^1^, ^2^, ^3^, ^4^, ^5^ A previous cohort study showed that light‐to‐moderate alcohol consumption during pregnancy was associated with a reduced risk of preterm delivery, regardless of the timing of exposure to alcohol.^6^ A recent meta‐analysis of 14 cohort and case–control studies also showed that light drinking of up to 18 g ethanol per day (1.5 drinks per day) was not linked to preterm delivery.^7^ On the other hand, although the information on the specific term of alcohol consumption during pregnancy was not included, a study composed of approximately 1 220 000 singleton records^8^ reported that prenatal alcohol consumption was positively associated with risk of preterm delivery. Several studies investigating the effects of light‐to‐moderate alcohol consumption have reported no association with preterm delivery.^9^, ^10^, ^11^, ^12^, ^13^, ^14^, ^15^

No firm conclusion with respect to the effect of alcohol consumption during pregnancy on the preterm delivery risk has been reached. Furthermore, no study has examined the effect of alcohol consumption at different stages of pregnancy on the risk of preterm delivery in a Japanese population.

Therefore, the association of alcohol consumption during the first trimester and during the second and third trimesters with preterm delivery risk was investigated using data from 94 349 pregnant women in a large birth cohort.

## Methods

### Study population

The Japan Environment and Children's Study (JECS), a nationwide birth cohort study, began in January 2011 and recruitment finished in March 2014. In total, 103 099 pregnancies were registered. Details concerning the JECS project have been presented in previous articles.^16^, ^17^ The JECS was approved by the Institutional Review Board of the Japan National Institute for Environmental Studies, as well as the ethics committees of all participating institutions. The present study is based on the jecs‐ag‐20160424 data set, which was released in June 2016 and revised in October 2016. Core outcome set and patient involvement are not relevant to this study. The JECS was funded by the Ministry of the Environment, Japan.^16^, ^17^

**Figure 1:**
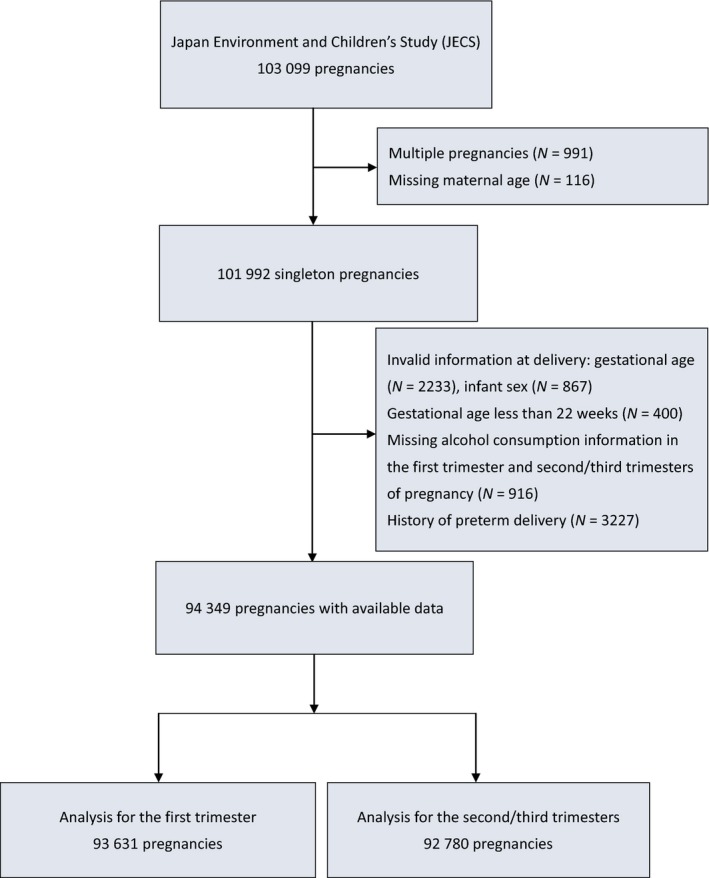
Participant selection flow chart.

Among the 101 992 singleton pregnant women eligible for the present study, we excluded 3100 individuals who had certain information, such as gestational age and infant sex, unavailable at delivery. An additional 400 women with an infant gestational age <22 weeks, 916 women with missing information on alcohol consumption for both stages of pregnancy and 3227 women with a history of preterm delivery were also excluded. We subsequently excluded pregnancies with missing alcohol consumption information at each stage of pregnancy. The analysis of the first trimester included 93 631 pregnancies, and 92 780 pregnancies were included in the analysis of the second and third trimesters. (Figure 1)

### Data collection

Self‐administered questionnaires were conducted during the first trimester and during the second and third trimesters. Information obtained included demographic characteristics, medical history, socio‐economic status, alcohol consumption, smoking, physical activity and psychological factors. Maternal anthropometric data before pregnancy as well as data on complications during pregnancy, medical history, history of previous pregnancies, infant sex and perinatal outcomes, such as gestational duration and birthweight, were collected through data transcription from medical records. Data transcription was performed by physicians, nurses, midwives or research coordinators. Dietary information was obtained with a food frequency questionnaire. Preterm delivery was defined as delivery between 22 and 37 weeks of gestation. We used participant weight before pregnancy to calculate maternal body mass index (weight [kg]/height^2^ [m^2^]).

We asked the participants whether they never drank, were past drinkers or were current drinkers to record alcohol consumption during the first trimester. Past and current drinkers were asked about the frequency of their alcohol consumption during the 1 year before pregnancy. They indicated whether they drank almost never, one to three times per month, once or twice per week, three or four times per week, five or six times per week or every day. We also recorded the amount of alcohol consumption per day according to the type of alcoholic beverage. Types of alcohol included *sake* (rice wine), *shochu awamori* (white spirits), beer, whisky and wine.

To assess alcohol consumption during the second and third trimesters, we asked participants whether they never drank, had quit drinking before pregnancy, had quit drinking during early pregnancy or currently drank. Current drinkers were then asked about the frequency and amount of their alcohol consumption per day as well as the type of alcoholic beverages consumed during pregnancy. We assigned fractions to alcohol consumption frequency categories as follows: 0 for almost never, 0.5 for one to three times per month, 1.5 for once or twice per week, 3.5 for three or four times per week, 5.5 for five or six times per week and 7 for participants drinking alcohol every day. The amount of ethanol was calculated in grams as follows: 180 ml *sake* has 23 g ethanol, 180 ml *shochu* or *awamori* has 36 g ethanol, 633 ml beer has 23 g ethanol, 30 ml whisky has 10 g ethanol and 60 ml wine has 9 g ethanol. Alcohol consumption per week was calculated by multiplying the frequency of drinking alcohol and the amount of ethanol per occasion according to the programme provided by the Japan Public Health Centre‐Based Prospective Study for the Next Generation.^18^ Participants were categorised according to alcohol consumption into non‐drinkers, current drinkers of 1–149 g ethanol per week, current drinkers of 150–299 g ethanol per week, and current drinkers of ≥300 g ethanol per week.

### Statistical analysis

Mean values and preterm delivery risk factor prevalence according to alcohol consumption were calculated. The odds ratios (ORs) with 95% CI for preterm delivery in relation to the alcohol consumption categories were estimated using logistic regression. These estimates were adjusted for age and other confounding factors including residential area (15 regional centres), smoking (never smoked, quit smoking before pregnancy, quit smoking during early pregnancy, currently smoking one to nine cigarettes per day or ten cigarettes or more per day), the frequency of passive smoking (almost never, 1 day/week, 2–6 days/week and every day), body mass index before pregnancy, education (junior high school, high school, technical college, vocational school, junior college, university or graduate school), physical activity (metabolic equivalent), history of hypertension and dietary folate intake (quintiles). We used updated smoking, physical activity and dietary folate intake data, which were obtained using the questionnaire for the second and third trimesters, in the analysis during pregnancy. We excluded participants with missing data related to outcome, exposure and biological factors such as maternal age and infant sex from the analysis. Missing data of confounding factors were included as categorical variables in the model. SAS 9.4 statistical software (SAS Institute Inc., Cary, NC, USA) was used for these analyses. We also used E‐value to conduct the sensitivity analysis for unmeasured confounding (E‐value calculator: https://www.evalue-calculator.com/). The E‐value estimates the minimum strength of the association of the unmeasured confounder with exposure and outcome to fully explain the observed association under the condition of the measured covariates.^19^

## Results

Table 1 shows characteristics based on alcohol consumption during the first trimester and during the second and third trimesters. In the first trimester, 91.2% of participants were non‐drinkers, 7.5% consumed 1–149 g ethanol per week, 0.8% consumed 150–299 g per week, and 0.5% consumed ≥300 g ethanol per week. The corresponding percentages of participants in each category during the second and third trimesters were 98.0% non‐drinkers, 1.96% consuming 1–149 g ethanol per week, 0.05% consuming 150‐299 g per week, and 0.03% consuming ≥300 g ethanol per week. The percentage of participants delivering preterm was 4.1% for the first‐trimester analysis and 3.4% for the analysis of the second and third trimesters.

**Table 1:** Mean and prevalence of characteristics according to alcohol consumption in the first trimester and in the second and third trimesters

	Non‐drinkers	Current drinkers (g ethanol/week)			*P* for trend
		1–149	150–299	≥300	
**Alcohol consumption in the first trimester**					
Persons	85 433	7011	761	426	
Age (years)	31.0	32.5	33.3	32.3	<0.001
Body mass index before pregnancy (kg/m^2^)	21.2	21.3	21.4	21.2	0.28
Education, university or graduate school (%)	21.2	29.3	20.8	14.9	0.43
Smoking in the first trimester (%)	4.7	3.6	11.0	29.1	<0.001
Passive smoking during pregnancy ≥1 day/week (%)	37.7	35.6	48.2	65.3	<0.001
Physical activity before pregnancy (METs*min/day)	410.1	359.3	425.6	474.5	0.26
Dietary folate intake for the past year (µg/day)	277.4	291.5	334.3	403.7	<0.001
Primiparity (%)	42.4	33.9	31.4	34.9	<0.001
Hypertensive disorders of pregnancy (%)	3.1	3.1	3.2	4.2	0.29
Gestational age at delivery (weeks)	38.9	38.8	38.9	38.6	0.003
Late preterm delivery (34 to <37 weeks) (%)	3.4	3.4	3.0	3.8	0.97
Moderate preterm delivery (32 to <34 weeks) (%)	0.4	0.4	0.3	0.5	0.99
Very preterm delivery (<32 weeks) (%)	0.6	0.6	0.6	1.4	0.09
Birthweight (g)	3027	3038	3022	2933	<0.001
Premature rupture of the membranes (%)	8.2	8.4	9.6	9.2	0.19
Spontaneous labour (%)	57.8	59.4	56.3	56.5	0.58
**Alcohol consumption in the second and third trimesters**					
Persons	90 893	1814	47	26	
Age (years)	31.1	32.3	33.8	30.4	<0.01
Body mass index before pregnancy (kg/m^2^)	21.2	21.2	21.5	20.9	0.92
Education, university or graduate school (%)	21.9	18.1	4.3	7.7	<0.001
Smoking in the second/third trimesters (%)	4.3	15.9	46.8	40.0	<0.001
Passive smoking during pregnancy ≥1 day/week (%)	37.5	51.4	74.5	60.0	<0.001
Physical activity during pregnancy (METs*min/day)	238.1	278.2	350.6	454.6	0.004
Dietary folate intake during pregnancy (µg/day)	258.3	278.0	306.0	345.3	<0.001
Primiparity (%)	42.1	20.0	6.5	30.8	<0.001
Hypertensive disorders of pregnancy (%)	3.1	2.5	8.5	11.5	0.003
Gestational age at delivery (weeks)	38.9	38.9	38.5	37.8	<0.001
Late preterm delivery (34 to <37 weeks) (%)	3.4	3.2	4.3	15.4	0.003
Moderate preterm delivery (32 to <34 weeks) (%)	0.4	0.2	2.2	0.0	0.54
Very preterm delivery (<32 weeks) (%)	0.5	0.1	0.0	3.8	0.11
Birthweight (g)	3031	3045	2925	2733	<0.001
Premature rupture of the membranes (%)	8.3	7.8	12.8	11.5	0.33
Spontaneous labour (%)	57.8	61.7	48.9	52.0	0.49

Drinkers of ≥300 g ethanol per week during the first trimester were older, more likely to be smokers, multiparous and exposed to passive smoking, and had higher folate intake and fewer gestational weeks and lower birthweight infants compared with non‐drinkers. Drinkers of ≥300 g ethanol per week during the second and third trimesters were younger, less likely to be educated, more likely to be smokers or exposed to passive smoking, more likely to have previous birth experience, hypertensive disorders of pregnancy, higher physical activity levels and folate intakes, and fewer gestational weeks and lower birthweight infants compared with non‐drinkers. Drinkers of ≥300 g ethanol per week during the second and third trimesters tended to have a higher incidence of late preterm delivery.

Table 2 shows the age‐adjusted and multivariable‐adjusted ORs (95% CI) for preterm delivery based on the categories of alcohol consumption in the first trimester and in the second and third trimesters. Alcohol consumption during the first trimester was not associated with increased risk of preterm birth, whereas alcohol consumption during the second and third trimesters showed a J‐shaped association with the risk of preterm delivery. Light drinking of 1–149 g ethanol per week was not significantly associated with reduced risk of preterm delivery, whereas heavy drinking of ≥300 g ethanol per week was associated with a significant increased risk of preterm delivery compared with non‐drinkers. Multivariable ORs were 0.78 (95% CI 0.60–1.00) and 4.52 (95% CI 1.68–12.2) for light drinking and heavy drinking, respectively.

**Table 2:** Odds ratios (OR) (95% CI) for preterm delivery according to alcohol consumption in the first trimester and in the second and third trimesters

	Non‐drinkers	Current drinkers (g ethanol/week)		
		1–149	150–299	≥300
**Alcohol consumption categories in the first trimester**				
Persons	85 433	7011	761	426
No. of cases	3745	300	30	24
Incidence of preterm delivery	4.4	4.3	3.9	5.6
Age‐adjusted OR	1.00	0.93 (0.83–1.05)	0.83 (0.58–1.20)	1.25 (0.83–1.89)
Multivariable OR*	1.00	0.97 (0.86–1.10)	0.81 (0.56–1.18)	1.05 (0.69–1.60)
**Alcohol consumption categories in the second/third trimesters**				
Persons	90 893	1814	47	26
No. of cases	3805	63	3	5
Incidence of preterm delivery	4.2	3.5	6.4	19.2
Age‐adjusted OR	1.00	0.79 (0.62–1.02)	1.44 (0.45–4.63)	5.58 (2.10–14.8)
Multivariable OR**	1.00	0.78 (0.60–1.00)	1.27 (0.39–4.12)	4.52 (1.68–12.2)

*Adjustment for maternal age, area, body mass index before pregnancy, education, smoking in the first trimester, frequency of passive smoking during the pregnancy, physical activity before pregnancy, primiparity and dietary folate intake for the year before pregnancy.

**Adjustment for maternal age, area, body mass index before pregnancy, education, physical activity during pregnancy, smoking in the second/third trimesters, frequency of passive smoking during the pregnancy, primiparity and dietary folate intake during pregnancy.

## Discussion

### Main findings

In the large birth cohort study, alcohol consumption during the first trimester was not associated with risk of preterm delivery, whereas in the second and third trimesters we found a J‐shaped association between alcohol consumption and risk of preterm delivery. Compared with non‐drinkers, heavy drinking (≥300 g ethanol per week) during the second and third trimesters had a three‐fold higher risk of preterm delivery, whereas light drinking (1–149 g ethanol per week) tended to be associated with the lower risk.

### Strengths and limitations

The main strengths of this study include that it was a large prospective study with a high response rate under a national birth cohort. We first found the association between a wide range of alcohol intakes during pregnancy and risk of preterm delivery in Asian women after adjustment for known confounding factors including passive smoking during pregnancy and folate intake and physical activity levels before or during pregnancy. We also examined the risk of preterm delivery associated with maternal alcohol consumption in different stages of pregnancy. The larger population‐based cohort study of 1 220 000 singletons linked to the Missouri vital statistical data^8^ examined retrospectively the association because alcohol consumption during pregnancy was measured at delivery and did not take the above confounding variables into account.

The present study also has several limitations. First, exposure misclassification may have occurred because the information on alcohol consumption during pregnancy was collected by self‐reported questionnaires. It is difficult to assess alcohol consumption among pregnant women because many pregnant women refrain from drinking alcohol after becoming aware of their pregnancy or as pregnancy progresses. We used the food frequency questionnaire validated in a general population of women, in which the Spearman's correlation coefficient for alcohol consumption between the food frequency questionnaire and 12‐day food records over four seasons at intervals of approximately 3 months was 0.67.^18^ As there was non‐differential exposure misclassification, the real association would become stronger. Second, we could not examine the associations of alcohol consumption patterns such as weekend or binge drinking during pregnancy with risk of preterm delivery because we did not collect that information. A previous study reported that binge drinking (five or more units per occasion) before pregnancy, during the first trimester or during the second trimester was not associated with an excess risk of preterm delivery.^9^ The Screening for Pregnancy Endpoints (SCOPE) study also showed no association between binge drinking before 15 weeks of gestation and risk of adverse pregnancy outcomes, including spontaneous preterm delivery.^10^ Third, east Asians are commonly intolerant of alcohol due to the larger prevalence of less active or inactive forms of aldehyde dehydrogenase‐2 encoded by ALDH2*1/*2 or ALDH2*2/*2 than that found in Caucasians.^20^ Therefore, east Asians are more likely to accumulate acetaldehyde and suffer from alcohol‐related diseases.^21^, ^22^, ^23^ However, the racial difference in alcohol metabolism did not meet the findings from previous^3^, ^8^ and present studies. Excess risk of preterm delivery was found among American women who consumed five or more drinks per week^8^ and European women who consumed ten or more drinks per week,^3^ and among Japanese women who consumed ≥28 drinks per week in the present study. Further studies will be necessary to accumulate epidemiological findings and evidence on mechanisms. Fourth, we did not examine the associations of spontaneous and medically indicated preterm deliveries separately because of the small number of heavy drinkers. The previous large study using the Missouri vital statistical data^8^ found increased risk of both outcomes associated with heavy drinking. Further work is needed for a detailed understanding of the effects of alcohol consumption on spontaneous and medically indicated preterm delivery. Finally, the associations were adjusted for potential confounding variables, but we cannot exclude the possibility that unmeasured confounding may have affected the findings. We conducted the sensitivity analysis for the unmeasured confounders by E‐value calculation. The calculated E‐value for point estimate was 8.51, so that the observed association would be unlikely to disappear with unmeasured confounders.

### Interpretation

Our finding of a J‐shaped relationship between alcohol consumption and risk of preterm delivery is consistent with the result from the Danish pregnancy cohort study.^3^ That Danish cohort study of 18 228 singleton pregnancies^3^ showed that ten or more drinks per week at 16 and 30 weeks of gestation produced 2.9 and 3.6 times higher risks of preterm delivery, respectively, whereas the consumption of one to two drinks per week at 30 weeks of gestation was associated with a 31% lower risk of preterm delivery compared with women who consumed less than one drink per week.

As for mechanisms of high alcohol consumption and risk of preterm delivery, alcohol may induce preterm delivery through increased secretion of prostaglandins, which enhances uterine contractions.^24^ Prostaglandins also increase cyclic 3ʹ,5ʹ‐adenosine monophosphate activity, which involves decreased cell division.^25^ In animal experiments, increased alcohol intake also induced increased severe intravascular coagulation^26^ and decreased blood flow in the placenta.^27^ According to a previous report from our cohort, alcohol consumption (≥150 g ethanol/week) in the second and third trimesters was associated with increased risk of hypertensive disorders of pregnancy, which is one of the risk factors for preterm delivery.^28^

There has been no conclusive biological mechanism proposed for the reduced risk of preterm delivery associated with light drinking. A previous study suggested the healthy drinker effect in explaining the association.^3^ In the present study, light drinkers during the second and third trimesters had fewer hypertensive disorders. The effect of light alcohol consumption during pregnancy should be interpreted cautiously in consideration of other adverse outcomes such as fetal alcohol syndrome, multiple morbidities and developmental disorders.

We found no association between alcohol consumption in the first trimester and risk of preterm delivery. The effects of different timings of alcohol consumption on the risk of preterm delivery remain controversial. The Danish pregnancy cohort study showed singleton mothers consuming ten or more drinks per week at 16 weeks of gestation was associated with increased risk of preterm delivery compared with those consuming less than one drink per week (OR 2.93, 95% CI 1.52–5.63), as with ten or more drinks per week at 30 weeks of gestation (OR 3.56, 95% CI 1.78–7.13).^3^ A case–control study of 175 singleton mothers with preterm deliveries and 313 singleton mothers with full‐term deliveries showed that 14 or more drinks per week in any trimester was associated with a three‐fold increase in the risk of preterm delivery based on interviews during the postpartum hospital stay.^29^ On the other hand, a prospective study consisting of 5628 nulliparous pregnant women (the SCOPE study) showed no association between consuming alcohol before 15 weeks of gestation with spontaneous preterm delivery or other adverse outcomes such as pre‐eclampsia and infants being small for their gestational age or having a low birthweight.^10^ A study of 3447 pregnant women in the Netherlands reported no association between alcohol consumption in each trimester and the risk of preterm delivery.^12^ Further study is necessary to determine the effects of alcohol consumption at different times during pregnancy on the risk of preterm delivery.

## Conclusion

In conclusion, heavy alcohol consumption in the second and third trimesters was associated with increased risk of preterm delivery among pregnant women.

### Disclosure of interests

None to declare. Completed disclosure of interest forms are available to view online as supporting information.

### Contribution to authorship

SI, TK, AK, TS and HI contributed substantially to the conception, design, participant recruitment and data collection of the present study. SI and TK were involved in data analysis. SI and HI drafted the manuscript. All authors were involved in interpreting the data and critically reviewing manuscript drafts. All authors gave approval of the final version of the manuscript.

### Details of ethics approval

The JECS was approved by the Institutional Review Board of the Japan National Institute for Environmental Studies (date of approval: 9 August 2010 and approval number: 2010‐2R), as well as the Ethics Committees of all participating institutions.

### Funding

The JECS was funded by the Ministry of the Environment, Japan (direct funding, no funding IDs available).

## Supporting information


** **

** **

** **

** **

** **

## Appendix 1

Members of JECS as of 2017 (principal investigator, Toshihiro Kawamoto): Hirohisa Saito (National Centre for Child Health and Development, Tokyo, Japan), Reiko Kishi (Hokkaido Regional Centre for JECS, Hokkaido University, Hokkaido, Japan), Nobuo Yaegashi (Miyagi Regional Centre for JECS, Tohoku University, Sendai, Japan), Koichi Hashimoto (Fukushima Regional Centre for JECS, Fukushima Medical University, Fukushima, Japan), Chisato Mori (Chiba Regional Centre for JECS, Chiba University, Chiba, Japan), Shuichi Ito (Kanagawa Regional Centre for JECS, Yokohama City University, Kanagawa, Japan), Zentaro Yamagata (Koshin Regional Centre for JECS, University of Yamanashi, Yamanashi, Japan), Hidekuni Inadera (Toyama Regional Centre for JECS, University of Toyama, Toyama, Japan), Michihiro Kamijima (Aichi Regional Centre for JECS, Nagoya City University, Aichi, Japan), Takeo Nakayama (Kyoto Regional Centre for JECS, Kyoto University, Kyoto, Japan), Hiroyasu Iso (Osaka Regional Centre for JECS, Osaka University, Osaka, Japan), Masayuki Shima (Hyogo Regional Centre for JECS, Hyogo College of Medicine, Hyogo, Japan), Yasuaki Hirooka (Tottori Regional Centre for JECS, Tottori University, Tottori, Japan), Narufumi Suganuma (Kochi Regional Centre for JECS, Kochi University, Kochi, Japan), Koichi Kusuhara (Fukuoka Regional Centre for JECS, Kyushu University, Fukuoka, Japan) and Takahiko Katoh (South Kyushu/Okinawa Regional Centre for JECS, Kumamoto University, Kumamoto, Japan).
